# Firework Optimization Algorithm-Based Diagnosis of Hepatocellular Carcinoma and Hepatic Cavernous Hemangioma Using MRI Images

**DOI:** 10.1155/2021/3970529

**Published:** 2021-07-21

**Authors:** Geng Liu, Huiqun Chen, Fang Fang, Lei Song

**Affiliations:** ^1^General Surgery, Tianjin Nankai Hospital, Tianjin 300000, China; ^2^Department Digestive Internal, The 967th Hospital of the Joint Logistics Support Force of People's Liberation Army of China, Dalian 116000, China; ^3^Hepatobiliary Surgery, Yantai Laiyang Central Hospital, Yantai 265200, China

## Abstract

This study was aimed to explore the diagnostic features of magnetic resonance imaging (MRI) on hepatocellular carcinoma (HCC) and hepatic cavernous hemangioma (HCH). A fireworks algorithm optimization (FAO) was proposed based on the fireworks algorithm (FA), and it was compared with the maximum between-class variance method (OTSU) and the maximum entropy threshold method (KSW) for analysis. In addition, it was applied to the diagnosis of MRI images of 55 HCC patients in the experimental group (group E) and 55 HCH patients in the control group (group C). It was found that the FAO showed a greatly lower difference function (DF) and a shorter running time in contrast to the OTSU and KSW algorithms (*P* < 0.05); the diagnostic accuracy (DA) of the T1-weighted image (T1WI) for patients in groups E and C was 85.31% and 95.85%, respectively, and the DA of the T2-weighted image (T2WI) was 97.84% (group E) and 89.71% (group C), respectively. In short, FAO showed an excellent performance in segmentation and reconstruction of MRI images for liver tissue, and T1WI and T2WI of MRI images showed high accuracy in diagnosing the HCC and HCH, respectively.

## 1. Introduction

HCC is one of the main malignant tumors causing cancer deaths worldwide and the main cause of death in patients with liver cirrhosis. It is the most common type of primary liver cancer [1, 2]. The most common clinical symptoms of HCC are liver pain, hepatomegaly, jaundice, signs of liver cirrhosis, systemic manifestations (such as weight loss, fever, loss of appetite, fatigue, and malnutrition), and even cachexia in severe cases [3]. There are many reasons for HCC, including various liver diseases, alcoholism, hepatitis B virus (HBV), and hepatitis C virus (HCV). HCH is the most common benign tumor, mainly due to the expansion and proliferation of capillaries in the liver or hepatic sinusoids. Academia generally believes that the occurrence of HCH is related to certain congenital factors, estrogen, certain drugs, and capillary tissue infections [4, 5]. HCH is often seen in B-ultrasound or during abdominal surgery, and there is no obvious clinical phenomenon in general patients. Only when the tumor is large or the tumor grows rapidly, will the abdominal mass, gastrointestinal discomfort, tumor rupture and bleeding, rapid growth of intratumoral bleeding, giant hemangioma, accompanied by thrombocytopenia and systemic bleeding tendency, and other related symptoms be found [6]. Therefore, if HCC and HCH are accurately identified, it is also a hotspot in clinical practice.

MRI is a new imaging technology developed in the 1980s. It can not only obtain detailed diagnostic images of human organs and tissues but also detect the chemical components and reactions of living organs and tissues. It is featured with clear images, high resolution, various parameters, and good soft tissue display [7, 8]. With the continuous development of technology in the medical field, high-end MRI technology based on conventional MRI has gradually been applied to the liver. Among them, diffusion-weighted image (DWI) focuses on the comparison of the dispersion of water molecules between tissues. It can obtain the characteristic information of the distribution and movement of water molecules in the living body and can be undertaken as a routine sequence for MRI scanning of the liver [9, 10]. The FA is inspired by the process of firework explosions producing sparks and continuing to split the explosion. It is mainly an explosion search mechanism suitable for global optimization and can be used as the solution space for optimization. In a feasible solution, the process of generating a certain number of sparks in the explosion is the process of searching the neighborhood [11]. Compared with genetic algorithm (GA), the FA can better avoid premature maturity, achieve excellent global convergence for high-dimensional functions, and has better adaptability [12]. Therefore, a new image segmentation and reconstruction algorithm was intended to be constructed in this study based on the FA to further explore the clinical diagnosis of HCC and HCH.

In summary, the FA is superior to the medical image evaluation. Based on this, an FAO was proposed by optimizing the FA, and it was applied in the MRI image diagnosis of 55 HCC patients in group E and 55 HCH patients in group C. The general information, lesion distribution, different sequence types, and DA of two groups of patients were compared so as to discuss the clinical characteristics of MRI images of HCC and HCH.

## 2. Materials and Methods

### 2.1. Selection of the Research Samples

110 patients who were admitted to the hospital for liver MRI examination from January 10, 2018, to February 15, 2020, were selected as the research objects, and they were divided into group E (with 55 HCC patients) and group C (with 55 HCH patients). In group E, there were 36 males and 19 females, with an age range of 19–64 years old; in group C, there were 35 males and 20 females, with an age range of 18–61 years old. The study had been approved by the Medical Ethics Committee of Hospital, and the patients and their families had understood the situation of the study and signed the informed consent forms.

The inclusion criteria could be defined as follows: patients diagnosed with HCC or HCH by surgical pathology; patients with evidence of liver cirrhosis; patients with clear consciousness and being able to cooperate with the examination; and patients with elevated alpha-fetoprotein caused by other causes (pregnancy, active liver disease, and secondary liver disease).

The exclusion criteria were defined as follows: patients younger than 18 years old; patients with mental illness; patients who had undergone surgery; patients with hypersensitivity to contrast agents; and patients with MRI examination contraindications.

### 2.2. MRI Examination

A new generation of 1.5T Signa HDxt nuclear MRI instrument from GE of the United States was used to examine the patient, equipped with an 8-channel phased array coil of the body produced by Jiangyin Wankang Medical Technology Co., Ltd. Before the scan, the patient was required to be in fasting and be without drinking water for 8 hours and undergo breathing training. The patient was placed in a supine position, with the feet in front, and the xiphoid was positioned. The scanning parameters were set as follows: the matrix was 128 × 128, the layer thickness was 2.5 mm, the layer spacing was 1.5 mm, the field of view was 30 × 30 cm, the time of repetition (TR) was 520 ms, and the time of echo (TE) was 35 ms. After the scan was completed, the image was sent to the workstation for image reconstruction and diagnostic analysis. The largest level with more uniform lesion signal was selected, the region of interest (ROI) within the lesion was determined, the lesion distribution position and different sequence signal types are measured, and DA was calculated.

### 2.3. Image Segmentation and Reconstruction Algorithm Based on the FA

The traditional threshold segmentation algorithm could obtain good image segmentation results, but it had the disadvantages of being susceptible to noise interference and unstable segmentation quality. The OTSU [13] was an algorithm to determine the image binarization segmentation threshold. Its calculation was simple and fast, and it was not affected by image brightness and contrast. Therefore, the gray level of the image was assumed to be [0,…, *H* − 1], and then the OTSU could be expressed as follows:

(1) $$\begin{array}{c} T_{i}=\frac{m_{i}}{m}, \end{array}$$

(2) $$\begin{array}{c} \sum_{i=0}^{H-1}T_{i}=1. \end{array}$$

In equation (1), *m*_*i*_ referred to the number of pixels with gray value *i*, and *m*=*m*_0_+*m*_1_+*m*_*H*−1_. *T*_*i*_ is the probability of gray value *i*. Then, the threshold *x* was introduced to divide the image into two categories, *R*_0_ and *R*_1_, which could be written as follows:

(3) $$\begin{array}{c} R_{0}=\left[0,1,\ldots ,x\right],\\ R_{1}=\left[x+1,x+2,\ldots .H-1\right]. \end{array}$$

The sum of the two possibilities could be calculated with the following equations:

(4) $$\begin{array}{c} V_{0}=\sum_{i=1}^{x}T_{i}=V_{x}, \end{array}$$

(5) $$\begin{array}{c} V_{1}=\sum_{i=x+1}^{H-1}T_{i}=1-V_{x}. \end{array}$$

In the above equations (4) and (5), *V*_0_ represented the sum of the probabilities of pixels under *R*_0_ and *V*_1_ represented the sum of the probabilities of *R*_1_ pixels. Next, *θ*_0_ and *θ*_1_ were introduced to represent the average value of the two categories of pixels and *θ*_*z*_ represented the total average value of the pixels of the image, and then following equations could be obtained:

(6) $$\begin{array}{c} \theta_{0}=\sum_{i=1}^{x}\frac{iT_{i}}{V_{0}}=\frac{\theta \left(x\right)}{V_{0}}, \end{array}$$

(7) $$\begin{array}{c} \theta_{1}=\sum_{i=x+1}^{H-1}\frac{iT_{i}}{V_{1}}=\frac{\theta_{Z}-\theta \left(x\right)}{V_{1}}, \end{array}$$

(8) $$\begin{array}{c} \theta_{Z}=\sum_{i=1}^{H-1}iT_{i}=V_{0}\theta_{0}+V_{1}\theta_{1}. \end{array}$$

In the above equations (6), (7), and (8), *θ*(*x*)=∑_*i*=1_^*x*^*iT*_*i*_. The variance of *R*_0_ and *R*_1_ could be expressed as equations (9) and (10), respectively:

(9) $$\begin{array}{c} \sigma_{0}^{2}=\sum_{i=1}^{x}\frac{{\left(i-V_{0}\right)}^{2}T_{i}}{V_{0}}, \end{array}$$

(10) $$\begin{array}{c} \sigma_{1}^{2}=\sum_{i=x+1}^{H-1}\frac{{\left(i-V_{1}\right)}^{2}T_{i}}{V_{1}}. \end{array}$$

In the above two equations, *σ*_0_^2^ represented the pixel variance of *R*_0_ and *σ*_1_^2^ referred to the pixel variance of *R*_1_. Similarly, the between-class and within-class variances *R*_0_ and *R*_1_ could be calculated with the following equations:

(11) $$\begin{array}{c} \sigma_{J}^{2}=V_{0}{\left(\theta_{0}-\theta_{Z}\right)}^{2}+V_{1}{\left(\theta_{1}-\theta_{Z}\right)}^{2}, \end{array}$$

(12) $$\begin{array}{c} \sigma_{N}^{2}=V_{0}\sigma_{0}^{2}+V_{1}\sigma_{1}^{2}. \end{array}$$

In equations (11) and (12) above, *σ*_*J*_^2^ and *σ*_*N*_^2^ represented the between-class variance and the within-class variance of *R*_0_ and *R*_1_, respectively. If threshold *x* could maximize the between-class variance and minimize the within-class variance, then *x* at this time would be the best threshold, which could be expressed as follows:

(13) $$\begin{array}{c} \overset{↔}{x}=\arg\;\max\left\{\frac{\sigma_{J}^{2}\left(x\right)}{\sigma_{N}^{2}\left(x\right)}\right\}. \end{array}$$

Therefore, solving the optimal threshold of segmented images was successfully concerted into finding the optimal value of the fitness function. The OTSU was more sensitive to the image noise, so the between-class variance function might have double peaks or multiple peaks when the target and background size ratio was very large. Therefore, the FA was introduced to optimize the steps, and the optimal solution was achieved through multiple iterations.

**Figure 1:**
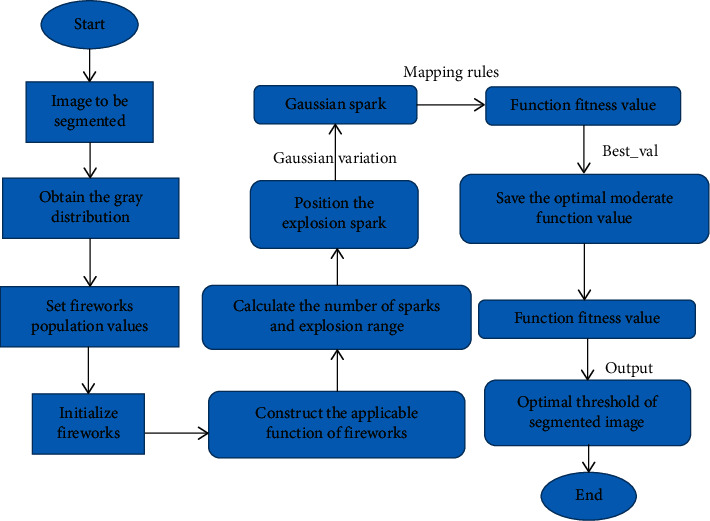
Processes for image segmentation and reconstruction based on the FA.

The operation process is shown in Figure 1. The medical image to be segmented was inputted, the gray value distribution was obtained, and the value range of the firework population was defined; a certain number of fireworks were initialized in the solution space, including the number of population and number of iterations, and the fitness function value of each firework was calculated; the number of sparks produced by each firework and the explosion range of the firework were obtained so as to determine the location of the explosion spark; the Gaussian spark was obtained with the Gaussian mutation based on the original firework. If the spark exceeded the boundary, the function fitness values of firework, explosion spark, and Gaussian spark were solved by mapping rules. The optimal fitness function value was saved with best_val, and the optimal threshold of the segmented image was outputted. Therefore, the image segmentation reconstruction algorithm was set based on FAO in this study.

### 2.4. Performance Evaluation Indicators of the Algorithms

The OTSU [14] and KSW [15] were introduced to compare with the FAO constructed in this study. The DF, the regional contrast (GC), and the running time were undertaken as the evaluation indicators. They could be calculated with following equations:

(14) $$\begin{array}{c} \text{DF}=\frac{\sum_{i=1}^{p}\sum_{j=1}^{q}\text{udf}\left(i,j\right)}{p\times q}, \end{array}$$

(15) $$\begin{array}{c} \text{udf}\left(i,j\right)=\left\{\begin{array}{c} 0,E\left(i,j\right)=E^{*}\left(i,j\right)\\ 1,E\left(i,j\right)\neq E^{*}\left(i,j\right) \end{array}\right\}, \end{array}$$

(16) $$\begin{array}{c} \text{GC}=\frac{\left|d_{1}-d_{2}\right|}{d_{1}+d_{2}}. \end{array}$$

In equations (14), (15), and (16), *E* represented the original image, *E*^*∗*^ represented the segmented image, and *p* × *q* referred to the size of the image. The value range of DF was [0, 1]. When the value was close to 0, it meant that the image had strong antinoise ability, and when it was close to 1, it meant that the image had weak antinoise ability. *d*_1_ represented the average gray value in a segmented area, and *d*_2_ represented the average gray value of adjacent areas of the segmented area. The GC value range was [0, 1]. The larger the value, the better the image segmentation effect.

### 2.5. Statistical Methods

The data processing was analyzed by SPSS19.0 version statistical software, the measurement data was indicated as mean ± standard deviation ($\bar{x}\pm s$), and the count data was displayed as percentage (%). The pairwise comparisons of DF, GC, and running time of FAO, OTSU, and KSW algorithms were realized with one-way analysis of variance. The age, height, weight, course of disease, ratio of male to female, number of cases of T1WI sequence signal, and the DA were compared in group E and group C by paired *t*-test. The difference was statistically meaningful at *P* < 0.05.

## 3. Results

### 3.1. Comparison of Diagnosis Performances of Three Algorithms

**Figure 2:**
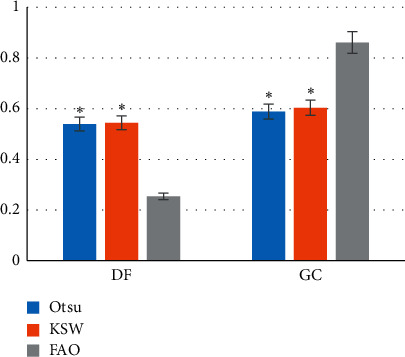
Comparison between DF and GC of three algorithms. *Note.*^*∗*^ indicates *P* < 0.05 in contrast to the FAO algorithm.

**Figure 3:**
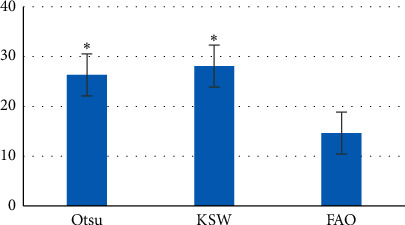
Comparison between running time of three algorithms. *Note.*^*∗*^ suggested *P* < 0.05 in contrast to the FAO algorithm.

Figures 2 and 3 show the comparisons of DF, GC, and running time of the three algorithms.  Figure 2 revealed that the DF and GC of the OTSU algorithm were 0.539 ± 0.084 and 0.588 ± 0.113, respectively; the DF and GC of the KSW algorithm were 0.544 ± 0.069 and 0.603 ± 0.074, respectively; and the DF and GC of the FAO were 0.254 ± 0.014 and 0.861 ± 0.102, respectively.  Figure 3 disclosed that the running time of OTSU, KSW, and FAO was 26.31 ± 6.96s, 28.06 ± 7.33s, and 14.62 ± 10.22s, respectively, of which, the DF and running time of the FAO were greatly shorter than those of the other two algorithms with obvious difference (*P* < 0.05); the GC of the FAO was higher obviously than that of the OTSU and KSW algorithms with observable differences (*P* < 0.05); and there was no dramatic difference in DF, GC, and running time of the OTSU algorithm and the KSW algorithm (*P* > 0.05).

### 3.2. Comparison of General Information of Two Groups of Patients

**Figure 4:**
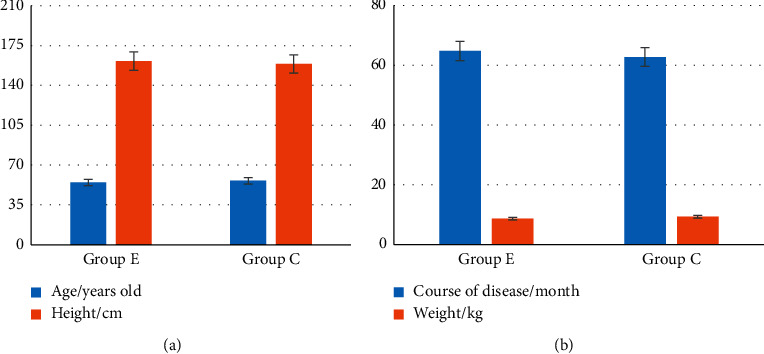
Comparison between age, height, weight, and disease duration of two groups of patients. (a) Comparison between age and height of patients. (b) Comparison between weight and course of disease of patients.

**Figure 5:**
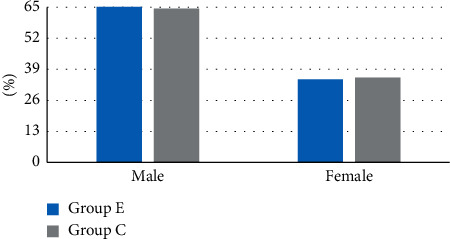
The ratio of male and female between the two groups.

Figure 4 shows the comparison of age, height, weight, and disease duration of two groups of patients. The ratio of male and female between the two groups is given in Figure 5. Figures 4 and 5 indicated that the age, height, weight, disease duration, and ratio of males and females of group E were not greatly different from those in group C (*P* > 0.05).

### 3.3. MRI Images of Some Patients before Surgery

**Figure 6:**
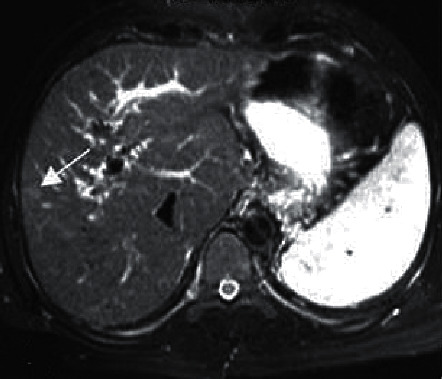
An MRI image of a male HCC patient (aged 50 years old).

**Figure 7:**
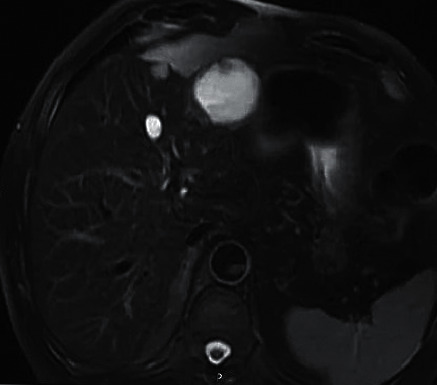
An MRI image of a male HCH patient (aged 61 years old).

Figure 6 shows an MRI image of a male HCC patient (aged 50 years old). It displayed that the T2WI sequence was limited; the HCC signal was slightly high; the tumor was visibly and uniformly enhanced in the arterial phase; the right lobe nodules were enlarged; the new, larger, and higher signal foci in the posterior lobe; the small foci showed isointensity; the tumor envelope of large foci was strengthened; and the right portal vein was invaded in the delayed phase.  Figure 7 shows an MRI image of a male HCH patient (aged 61 years old). The liquid level showed two different signals on the upper and lower layers and the point-like enhancement at the edge of the tumor strengthened towards the center over time. Except for the cystic degeneration, the entire tumor parenchyma was strengthened, and the density or intensity was uniform.

### 3.4. Comparison of Lesion Distribution of Patients in Two Groups

**Figure 8:**
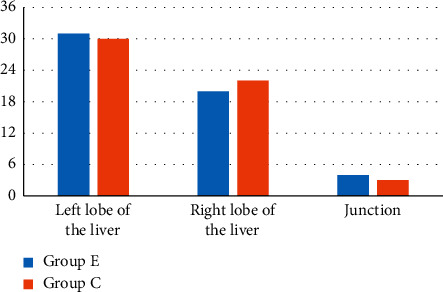
Comparison of lesion distribution of patients in two groups.

The distribution of lesions between the two groups was compared, as shown in Figure 8. It illustrated that there were 31 patients with lesions in the left lobe, 20 cases with the lesions in right lobe, and 4 cases with lesions at the junction of the left and right lobes in group E; there were 30 cases with lesions in the left lobe, 22 cases with lesions in the right liver, and 3 cases with lesions at the junctions in group C. Among them, the number of cases of with lesions in the left liver, right liver, and junction in the group E was not extremely visible in contrast to those in group C (*P* > 0.05).

### 3.5. Comparison of MRI Signal and Boundary Definition of Lesions in Patients between Two Groups

**Figure 9:**
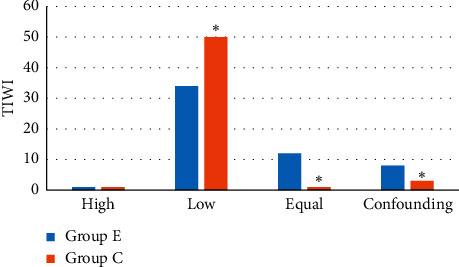
Comparison of T1WI sequence signal levels for patients between the two groups. *Note.*^*∗*^ indicates visible difference in contrast to group E (*P* < 0.05).

Figure 9 shows the comparison of T1WI sequence signal levels for patients between the two groups. It revealed that there was 1 case of T1WI high signal, 34 cases of low signal, 12 cases of equal signal, and 8 cases of confounding signal in group E; there was 1 case of T1WI high signal, 50 cases of low signal, 1 case of equal signal, and 3 cases of confounding signals in group C. Among them, the number of T1WI sequence low signal cases in group E patients was much less than that in group C (*P* < 0.05); the number of cases with T1WI equal signal and confounding signal in group E patients was dramatically more in contrast to group C (*P* < 0.05); and the number of patients with T1WI sequence high signal in the two groups was not extremely visible (*P* > 0.05).

**Figure 10:**
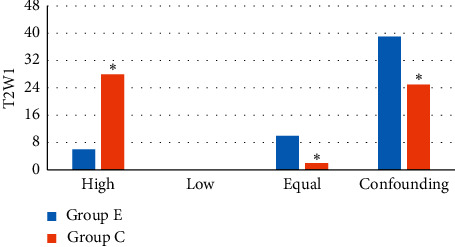
Comparison on T2WI sequence signal levels in patients between the two groups. *Note.*^*∗*^ indicates visible difference in contrast to group E (*P* < 0.05).

Figure 10 shows the comparison of T2WI sequence signal levels in patients between the two groups. It revealed that there were 6 cases of T2WI high signal, 0 cases of low signal, 10 cases of equal signal, and 39 cases of confounding signal in group E; there were 28 cases of T2WI high signal, 0 cases of low signal, 2 cases of equal signal, and 25 cases of confounding signals in group C. Among them, the number of T2WI sequence high signal cases in group E patients was much less than that in group C (*P* < 0.05); the number of cases with T2WI equal signal and confounding signal in group E patients was dramatically more in contrast to group C (*P* < 0.05), and the numbers of patients with T2WI sequence low signal in the two groups were not extremely visible (*P* > 0.05).

**Figure 11:**
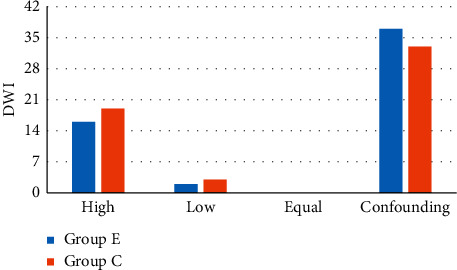
The comparison of DWI sequence signal levels between the two groups of patients.

Figure 11 reveals the comparison of DWI sequence signal levels between the two groups of patients. It disclosed that there were 16 cases with DWI sequence high signal, 2 cases with low signal, 10 cases with equal signal, and 37 cases with confounding signal in group E, and there were 19 cases of DWI sequence high signal, 3 cases of low signal, 0 cases of equal signal, and 33 cases of confounding signals in group C. Among them, the number of DWI sequence high, low, equal, and confounding signal cases of group E patients was not extremely different from that in group C (*P* > 0.05).

### 3.6. Comparison on DAs of Two Groups of Patients Based on T1WI and T2WI

**Figure 12:**
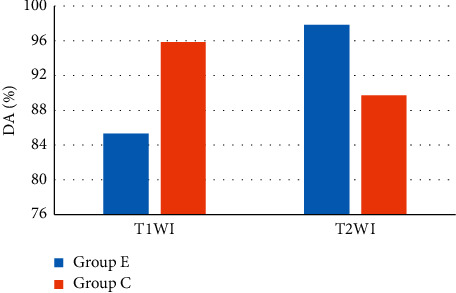
Comparison on DAs of two groups of patients based on T1WI and T2WI.

Comparison of DAs of two groups of patients based on T1WI and T2WI is given in Figure 12. It suggested that the DA of T1WI in groups E and C was 85.31% and 95.85%, respectively; while the DA of T2WI in groups E and C was 97.84% and 89.71%, respectively.

## 4. Discussion

HCC and HCH are relatively common benign and malignant tumors of the liver. Among them, HCC has the characteristics of high morbidity and high mortality and is one of the main causes of death from tumor diseases. HCH often has no obvious symptoms, and early clinical diagnosis is difficult [16]. Therefore, an image segmentation reconstruction algorithm FAO was proposed based on optimization of FA, and the OTSU and KSW were introduced for comparative analysis. It was found that the DF and running time of the FAO were much shorter in contrast to the Otsu and KSW algorithms, and the GC was much higher (*P* < 0.05). It was similar to the results of Xu et al. [17], indicating that the FAO showed excellent performance in segmentation and reconstruction of MRI images of liver tissues.

The FAO constructed was applied to the MRI image diagnosis of 55 HCC cases and 55 HCH cases. The results showed that the number of patients with lesions at the left, right, and junction of two liver lobes was not so different with that in group C (*P* > 0.05), indicating that the distribution of HCC and HCH showed no great difference. The number of cases with T1WI sequence low signal in group E was much less, and the number of patients with equal and confounding signal was extremely more (*P* < 0.05). Such results were consistent with the results of Puhr-Westerheide et al. [18], so it was speculated that the T1WI sequence equal and confounding signals could be applied for the diagnosis of HCC, and the T1WI sequence low signal could be applied for the diagnosis of HCH. The number of patients with T2WI sequence high signal in group E was less, and the number of equal and confounding signals was more visible. It indicated that the T2WI sequence equal and confounding signals could be applied for the diagnosis of HCC, while its high signal could be utilized for the diagnosis of HCH [19]. The DA of the T1WI sequence signal for groups E and C patients was 85.31% and 95.85%, respectively; while the DA of T2WI sequence signal for the groups C and E was 89.71% and 97.84%, respectively. It was similar to the research results of Esposito et al. [20], indicating that T1WI and T2WI in the MRI image showed high DAs in HCH and HCC, respectively.

In summary, the new image segmentation reconstruction model constructed by the FA was very helpful to improve the quality of MRI images and is helpful for assisting physicians in the interpretation of MRI images. In addition, different MRI sequences showed various evaluation effects for HCC and HCH. T1WI was more suitable for the clinical diagnosis of HCH, and T2WI was more suitable for the clinical diagnosis of HCC. Finally, it could be seen that MRI imaging based on the optimized FA image segmentation model had good application value in the diagnosis of HCH and HCC.

## 5. Conclusion

An image segmentation reconstruction algorithm FAO was proposed based on optimization of FA, and it was compared with the OTSU and KSW algorithms. In addition, it was applied in the MRI image diagnosis of 55 HCC patients and 55 HCH patients. The results found that the FAO proposed showed excellent performance in segmentation and reconstruction of liver tissue MRI images. T1WI and T2WI of MRI images showed high accuracy in the diagnosis of HCC and HCH. Among them, T1WI could achieve a better diagnosis effect for HCH, and T2WI could achieve a better diagnosis effect for HCC. However, the sample size of patients selected was too small, and there was a certain deviation. In the follow-up, it will consider increasing the sample size of patients and further exploring the application value of FA in liver cancer imaging diagnosis. In short, the results of this study could provide a theoretical basis for the clinical diagnosis of HCC and HCH.

## Data Availability

No data were used to support this study.
